# Red Macroalgae as Sources of Antioxidant and Multifunctional Ingredients for Functional Foods: A Biorefinery Approach

**DOI:** 10.3390/md24040145

**Published:** 2026-04-21

**Authors:** Nuno Nunes, Emmanuel Nunes, Kilian Odenthal, Miguel Â. A. Pinheiro de Carvalho

**Affiliations:** 1 ISOPlexis Centre for Sustainable Agriculture and Food Technology, Campus da Penteada, University of Madeira, 9020-105 Funchal, Portugal; emmanuel.nunes@staff.uma.pt (E.N.); kilian.odenthal@staff.uma.pt (K.O.); miguel.carvalho@staff.uma.pt (M.Â.A.P.d.C.); 2 Centre for the Research and Technology of Agroenvironmental and Biological Sciences, CITAB, Inov4Agro, Universidade de Trás-os-Montes e Alto Douro, UTAD, Quinta de Prados, 5000-801 Vila Real, Portugal; 3 Faculty of Life Sciences, Campus da Penteada, University of Madeira, 9020-105 Funchal, Portugal

**Keywords:** macroalgae, biorefinery, antioxidant activity, cholinesterase inhibition, α-glucosidase inhibition, functional extracts

## Abstract

Given the established interplay between oxidative stress, cholinergic dysfunction, and metabolic imbalance in cognitive decline, this study investigated the multifunctional potential of three red macroalgae from the Madeira Archipelago (*Asparagopsis taxiformis*, *Grateloupia lanceola*, and *Nemalion elminthoides*) using a sequential biorefinery approach. Marine algae represent a sustainable source of functional food ingredients due to their rich content in bioactive compounds and their compatibility with low-impact production systems. Protein, ethanolic (phenolic-rich), and polysaccharide fractions were obtained through direct extraction and scalable biorefinery processing. Antioxidant activity was evaluated using ORAC, DPPH, FRAP, and FIC assays, while functionality relevant to human health was assessed through acetylcholinesterase, butyrylcholinesterase, and α-glucosidase inhibition. Protein extracts, particularly from *N. elminthoides*, exhibited strong hydrogen atom transfer-based antioxidant capacity, whereas ethanolic extracts demonstrated multifunctional activity, combining radical scavenging, metal chelation, and enzyme inhibition associated with neuroprotective and glycemic-regulation potential. Polysaccharide fractions contributed mainly to iron chelation and reducing capacity. Correlation analyses highlighted the complementary nature of antioxidant and bioactivity assays. Overall, these findings support the potential of Madeira red macroalgae as functional food ingredients and emphasize the importance of optimized biorefinery strategies to maximize nutritional and health-related benefits.

## 1. Introduction

Macroalgae are a largely untapped source of bioactive compounds with increasing relevance for the development of sustainable functional foods and ocean-based nutrition systems. Unlike terrestrial crops, they do not compete for arable land or freshwater. Classified into brown (*Phaeophyceae*), red (*Rhodophyta*), and green (*Chlorophyta*) groups, macroalgae produce antioxidants [1] to withstand harsh marine conditions, directly shaping their biochemical composition [2,3,4]. To enhance economic feasibility and minimize waste, efficient extraction and purification methods must be developed to obtain multiple compounds from macroalgal biomass. Integrating these processes into a biorefinery framework enables the production of food-grade functional ingredients while supporting a low-carbon and sustainable economy [5]. However, research specifically addressing the extraction and purification of red macroalgae-derived products remains limited due to their unique composition [2,3,6]. Exposure to extreme environmental factors such as intense light, salinity fluctuations, oxygen variations, and nutrient scarcity induces oxidative stress in macroalgae. In response, they have evolved adaptive mechanisms to produce secondary metabolites that enhance resilience [7]. In red macroalgae, phycobiliproteins, known to contribute to antioxidant activity and nutritional value, are the most important light-harvesting complex, mainly composed of R-phycoerytrin, a water-soluble red pigment, which already demonstrated antioxidant capacity, extracted from *Gelidium sesquipedale*, collected in the French Basque Coast [8]. Polyphenols serve as key antioxidants, offering significant nutraceutical and functional food potential [9]. These are usually extracted using water [10], and organic solvents such as methanol [9] and ethanol [11]. These compounds have been shown to regulate glycemic responses by inhibiting digestive enzymes like α-amylase and amyloglucosidase, thereby reducing postprandial blood sugar spikes [12]. Also, Mycosporine-like amino acids (MAA) were found in 39 red macroalgae collected in Brazil [13] and these are also known for their high antioxidant activity [14] that could potentially increase the applicability of their extracts. Polysaccharides also exhibit antioxidant properties, influenced by their sugar ring organization, solubility, molecular weight, functional group composition, protein fractions, and the presence of covalently linked phenolics [15]. Several antioxidant assays were employed to identify the most promising extracts and the dominant antioxidant mechanisms.

Cognitive decline and impaired cholinergic function are major concerns associated with aging, and dietary strategies targeting cognitive health have gained increasing attention [16]. We have previously published the anticholinergic capability of macroalgae extracts in vitro [17] and other studies have also published results from in vivo models [18]. These assessments contribute to the identification of dietary compounds capable of supporting cholinergic function and cognitive health. In this work, ethanolic extracts were further studied in their potential as anticholinergic and antiglucosidase to determine further applications for these phenolic extracts. Oxidative stress is a central hallmark of Alzheimer’s disease (AD), contributing to neuronal damage, mitochondrial dysfunction, and amyloid-β (Aβ) aggregation. Redox-active metals such as iron and copper have been shown to catalyze reactive oxygen species (ROS) formation and promote Aβ aggregation, exacerbating neurotoxicity [19,20]. Therefore, metal chelation strategies may represent an important complementary mechanism for modulating neurodegenerative progression. In this context, inhibition of α-glucosidase, a key enzyme involved in carbohydrate digestion, represents a dietary strategy to attenuate postprandial glucose spikes and reduce systemic oxidative burden. This mechanism may complement direct antioxidant and cholinesterase-targeted activities within an integrated neuro–metabolic modulation framework [21]. Growing evidence supports a strong link between metabolic dysregulation and neurodegeneration. Insulin resistance and chronic hyperglycemia contribute to oxidative stress and advanced glycation end-product (AGE) formation, processes closely associated with Alzheimer’s disease pathology [22,23,24,25]. These interconnected mechanisms support the concept of a metabolic–neurodegenerative axis underlying cognitive decline.

Despite the recognized bioactive potential of red macroalgae, an integrated evaluation combining antioxidant profiling, enzyme inhibition, and scalable biorefinery processing remains limited. Therefore, the present study investigates fraction-specific extracts from *Asparagopsis taxiformis*, *Grateloupia lanceola*, and *Nemalion elminthoides* collected in the Madeira Archipelago, aiming to assess their capacity to modulate interconnected oxidative, cholinergic, and metabolic pathways within a sustainable biorefinery framework.

## 2. Results

**Figure 1:**
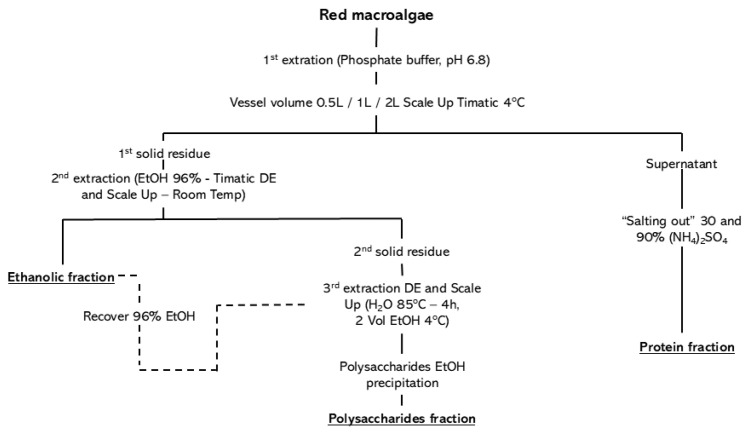
Sequential biorefinery workflow applied to red macroalgae, showing scale-up aqueous extraction (0.5, 1, and 2 L) followed by protein recovery via ammonium sulfate precipitation, ethanolic extraction of the solid residue, and subsequent polysaccharide extraction and precipitation. Final extracts for analysis are marked in bold and underlined. The path of ethanol recovery, a by-product of the ethanolic extract, is marked with dashed lines.

Following the sequential biorefinery workflow illustrated in Figure 1, this includes scale-up extraction steps (0.5, 1, and 2 L) and the successive recovery of protein, ethanolic, and polysaccharide fractions. This section presents the results of antioxidant activity assessment (Section 2.1), bioactivity evaluation (Section 2.2) and statistical analysis (Section 2.3).

### 2.1. Antioxidant Activities

#### 2.1.1. Oxygen Radical Absorbance Capacity (ORAC)

**Figure 2:**
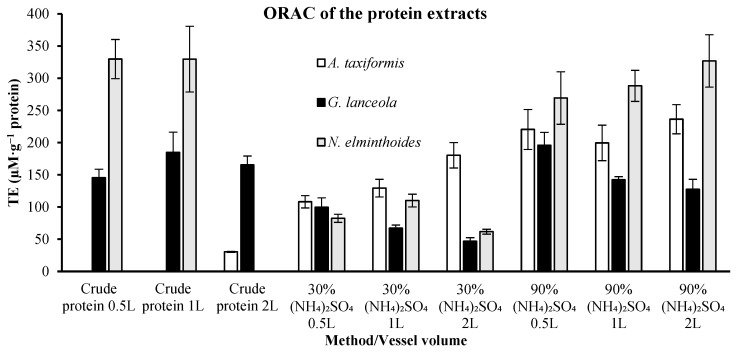
Assessment of the Oxygen Radical Absorbance Capacity (ORAC) in Trolox equivalents (TE) of the protein extracts, presenting the values in µM per gram (g) of protein in the extract.

The antioxidant capacity (ORAC) was assessed in the protein, ethanolic and polysaccharide extracts derived from the implementation of the biorefinery assessment. Protein ORAC assay from crude protein extracts of the macroalgae *A. taxiformis* shows that results (Figure 2) were negligible in the first two vessel volumes and reached a value of 30.38 ± 0.84 µM TE.g^−1^ of protein for the 2 L vessel.

Higher ORAC values varying between 108.09 ± 9.58 to 236.39 ± 22.68 µM TE.g^−1^ of protein were obtained when the extracts obtained by 30 to 90% ammonium sulphate precipitation were assessed. However, scale-up effects were also observed, as ORAC values varied according to vessel volume (0.5, 1, and 2 L), indicating that both fractionation level and extraction scale influence antioxidant capacity. The crude protein extracts of macroalgae *G. lanceola* have values of antioxidant capacity between 145.51 ± 13.31 and 184.93 ± 31.38 µM TE.g^−1^ of protein, and did not improve with the 30 to 90% ammonium sulphate precipitation, 46.75 ± 5.59 to 195.86 ± 20.12 µM TE.g^−1^ of protein, respectively. The macroalgae *N. elminthoides* presented between 0 and 329.73 ± 51.01 µM TE.g^−1^ of protein in the crude protein extracts and did not improve with protein concentrated by precipitation in the interval of 30 to 90% ammonium sulphate, 61.76 ± 3.78 to 326.97 ± 40.66 µM TE.g^−1^ of protein, respectively.

**Figure 3:**
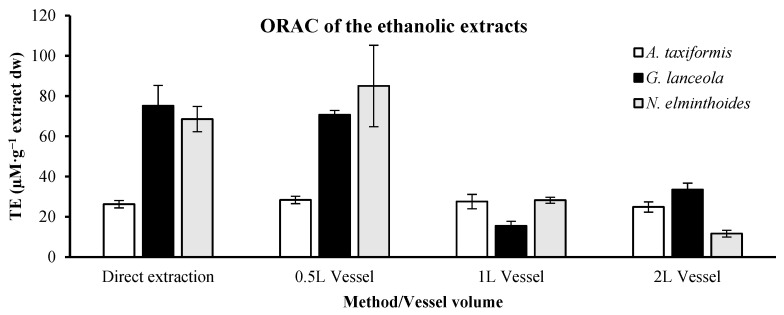
Assessment of the Oxygen Radical Absorbance Capacity (ORAC) in Trolox equivalents (TE) of the ethanolic extracts, presenting the values in µM per gram (g) of extract dry weight (dw).

The highest ORAC value for the *A. taxiformis* ethanolic extract (Figure 3), assessing the biorefinery sequential residue, was derived from the lower volume vessel (0.5 L), 28.38 ± 1.84 µM TE.g^−1^ extract dw, slightly higher than the values obtained from the direct extraction, 26.26 ± 1.83 µM TE.g^−1^ extract dw. For *G. lanceola*, the highest value for ORAC, applying the integrated fractionation strategy, was also obtained with the 0.5 L vessel, 35.35 ± 2.16 µM TE.g^−1^ extract dw, but the direct extraction allowed us to obtain 75.17 ± 10.15 µM TE.g^−1^ extract dw. The *N. elminthoides* presented the highest values obtained, 85.01 ± 10.11 µM TE.g^−1^ extract dw, with the 0.5 L vessel, within the direct method, rendering a lower value, 68.56 ± 6.30 µM TE.g^−1^ extract dw.

**Figure 4:**
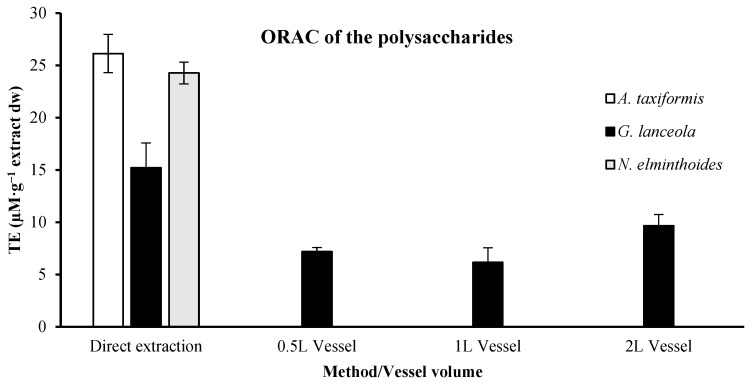
Assessment of the Oxygen Radical Absorbance Capacity (ORAC) in Trolox equivalents (TE) of the polysaccharides extract, presenting the values in µM per gram (g) of extract dry weight (dw).

ORAC values for polysaccharide extracts were only determined for *G. lanceola*, for which both direct and biorefinery-derived extracts (0.5, 1, and 2 L) were available (Figure 4). For *A. taxiformis* and *N. elminthoides*, ORAC values could only be assessed in the direct polysaccharide extracts, as no polysaccharide fractions were obtained following the sequential biorefinery process. Comparing the ORAC values for direct extractions, *A. taxiformis* presented the highest value, 26.13 ± 1.83 µM TE.g^−1^ extract dw. The *G. lanceola* presented values in an interval between 6.16 ± 1.40 and 9.67 ± 1.07 µM TE.g^−1^ extract dw with the integrated fractionation strategy, lower than the values obtained by direct extraction, 15.22 ± 2.36 µM TE.g^−1^ extract dw.

#### 2.1.2. Free Radical Scavenging Assay (DPPH)

**Figure 5:**
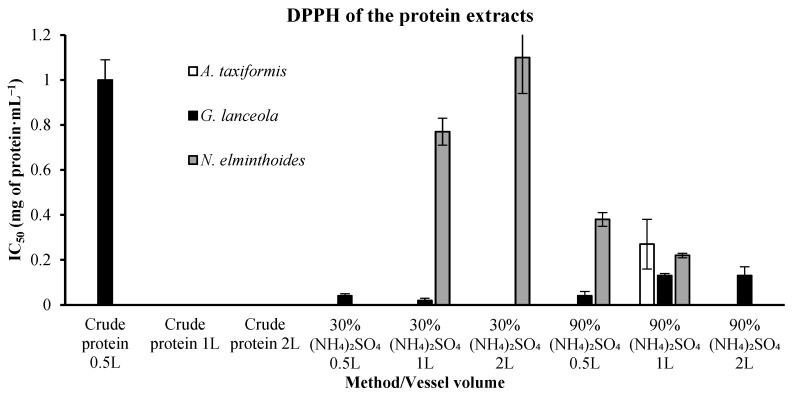
DPPH radical scavenging activity of protein-derived extracts from the red macroalgae evaluated in this study. IC_50_ values (mg·mL^−1^), representing the concentration required to inhibit 50% of the stable radical 2,2-diphenyl-1-picrylhydrazyl. (DPPH), are shown for crude protein extracts and ammonium sulfate-fractionated extracts obtained at different vessel volumes. Bars represent mean values ± SD of independent measurements. (NH_4_)_2_SO_4_—ammonium sulfate. Ascorbic acid, used as a positive control, exhibited an IC_50_ value of 0.00811 mg·mL^−1^.

This assessment was carried out in all the biorefinery extracts, through the comparison of the 50% inhibitory concentration (IC_50_ mg·mL^−1^) needed to prevent oxidation in a stable radical. For this test, lower values are preferable since it demonstrates that less extract concentration is needed to reach the 50% inhibitory activity. Assessment of the protein extracts (Figure 5) for *A. taxiformis* was only possible with the 90% ammonium sulphate precipitation in the 1 L vessel, demonstrating a low IC_50_ (0.27 ± 0.11 mg·mL^−1^). The only crude extract that was possible to quantify derived from *G. lanceola* and showed a result of 1.00 ± 0.09 mg·mL^−1^. The 30% ammonium sulphate precipitation extracts show a very low IC_50_ for *G. lanceola*, with 0.02 ± 0.01 (1 L vessel) and 0.04 ± 0.01 mg·mL^−1^ (0.5 L vessel). Still, the 90% ammonium sulphate precipitation extracts of *G. lanceola* exhibited good results, varying from 0.04 ± 0.02 (0.5 L vessel) to 0.13 ± 0.04 mg·mL^−1^ (2 L vessel). The *N. elminthoides* developed a low IC_50_ result within the 90% ammonium sulphate precipitation extracts, with 0.38 ± 0.03 and 0.22 ± 0.01 mg·mL^−1^, for the 0.5 L and 1 L vessels, respectively.

**Figure 6:**
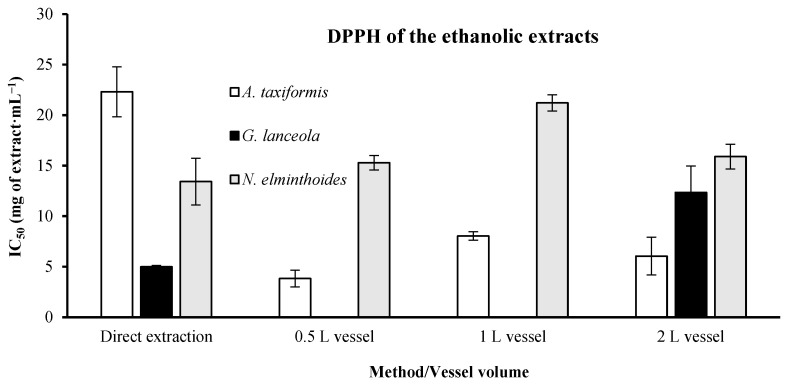
DPPH radical scavenging activity of ethanolic extracts obtained through direct extraction and sequential biorefinery processing at different vessel volumes. IC_50_ values (mg·mL^−1^), representing the concentration required to inhibit 50% of the stable radical 2,2-diphenyl-1-picrylhydrazyl (DPPH), are shown for each extraction condition. Bars represent mean values ± SD of independent measurements. Ascorbic acid, used as a positive control, exhibited an IC_50_ value of 0.00811 mg·mL^−1^.

The *A. taxiformis* ethanolic extracts (Figure 6) from the direct extraction demonstrated an IC_50_ value of 22.30 ± 2.47 mg·mL^−1^, higher than the best result found using the integrated fractionation strategy, where the lowest IC_50_ value, 3.82 ± 0.83 mg·mL^−1,^ was obtained using the 0.5 L vessel. The direct extract of *G. lanceola* had an IC_50_ concentration of 4.98 ± 0.13 mg·mL^−1^. The biorefinery extracts only reached an IC_50_ of 12.34 ± 2.63 mg·mL^−1^ obtained from the 2 L vessel. The direct extract of *N. elminthoides* had an IC_50_ of 13.42 ± 2.31 mg·mL^−1^, very similar to the best result of the biorefinery extracts, in which the lowest IC_50_, 15.28 ± 0.71 mg·mL^−1^, was obtained in the 0.5 L vessel.

**Figure 7:**
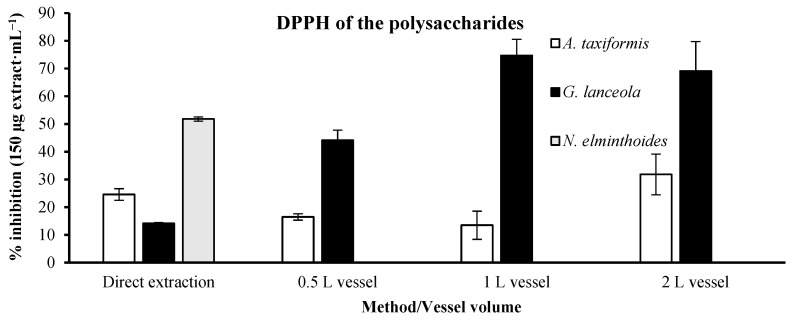
DPPH radical scavenging activity of polysaccharide extracts obtained through direct extraction and sequential biorefinery processing at different vessel volumes. The percentage of inhibition (%) was determined at a fixed concentration of 150 µg·mL^−1^ using the stable radical 2,2-diphenyl-1-picrylhydrazyl (DPPH). Bars represent mean values ± SD of independent measurements.

It was not possible to ascertain the IC_50_ results for the inhibitory activity using the polysaccharide extracts (Figure 7). Alternatively, the inhibitory activity was assessed at 150 µg·mL^−1^ concentration, to make a feasible comparison. The percentage of inhibition assessed in the direct extract of *A. taxiformis* was within (24.58 ± 2.09%) the subsequent biorefinery extracts, which presented an interval of variation between 13.49 ± 5.10 and 31.82 ± 7.35%. This was not the case with *G. lanceola*, where the percentage of inhibition was lower (14.14 ± 0.27%) when compared with the biorefinery extracts (44.13 ± 3.65 to 74.71 ± 5.87%). *Nemalion elminthoides* direct extract was the only one assessed for this macroalgae, 51.77 ± 0.75% of inhibition, due to the impossibility of extracting the polysaccharide from its residue.

#### 2.1.3. Ferric Reducing Antioxidant Power (FRAP)

**Figure 8:**
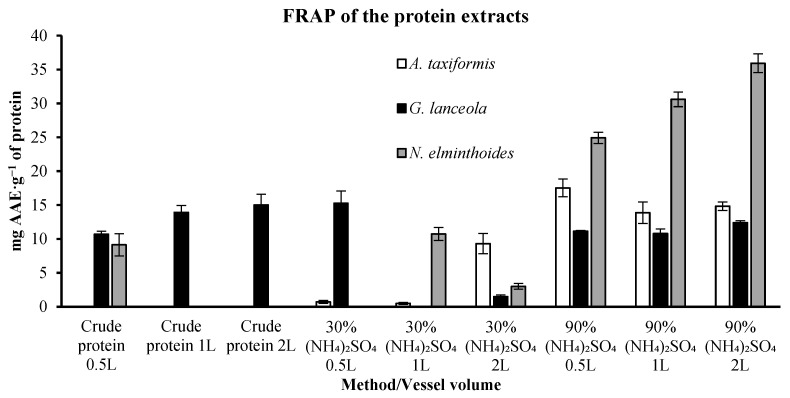
Ferric Reducing Antioxidant Power (FRAP) of protein-derived extracts obtained through direct extraction and sequential biorefinery processing at different vessel volumes. Results are expressed as mg of ascorbic acid equivalents per gram of protein (mg AAE·g^−1^ protein). Bars represent mean values ± SD of independent measurements.

This evaluation was performed in all the direct and biorefinery extracts derived from protein, ethanolic and polysaccharides. FRAP results for protein fractions are presented in Figure 8. *A. taxiformis* demonstrated a maximum value of 17.53 ± 1.31 mg of Ascorbic Acid Equivalents (AAE) per gram (g) of protein in the extract, obtained within the extract derived from the 90% ammonium sulphate precipitation, initially using the 0.5 L vessel on the Timatic equipment. For *G. lanceola*, the highest AAE concentration was observed in the 30% (NH_4_)_2_SO_4_ protein extract, derived from the 0.5 L vessel, 15.27 ± 1.80 mg AAE.g^−1^ of protein, decreasing the value as vessel volume increased. The *N. elminthoides* extracts derived from the 90% ammonium sulphate precipitation showed the highest value recorded in this work, proportional with the vessel volume used, varying from 24.92 ± 0.83 using the 0.5 L vessel to 35.93 ± 1.38 mg AAE.g^−1^ of protein using the 2 L vessel.

**Figure 9:**
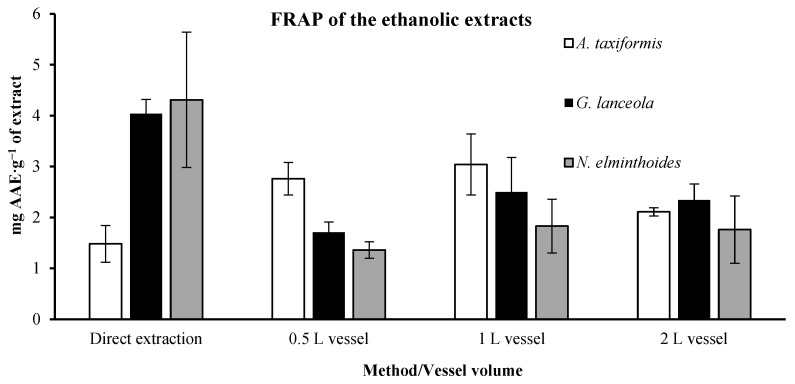
Ferric Reducing Antioxidant Power (FRAP) of ethanolic extracts obtained through direct extraction and sequential biorefinery processing at different vessel volumes. Results are expressed as mg of ascorbic acid equivalents per gram of extract (mg AAE·g^−1^ extract). Bars represent mean values ± SD of independent measurements.

The results of AAE in ethanolic extracts are given in Figure 9. The direct extract of the *A. taxiformis* holds a lower AAE value (1.48 ± 0.36 mg AAE.g^−1^ of extract) than the extracts derived from the residual biomass. The highest value was reached using the 1 L vessel (3.04 ± 0.60 mg AAE.g^−1^ of extract). However, this was not the case for *G. lanceola* and *N. elminthoides*, showing higher values in the direct extract, 4.04 ± 0.28 and 4.31 ± 1.33 mg AAE.g^−1^ of extract, respectively. These values decreased to 2.50 ± 0.68 and 1.83 ± 0.53 mg AAE.g^−1^ of extract when using the 1 L vessel, which were the highest values detected in the extracts derived from the residual biomass in these macroalgae.

**Figure 10:**
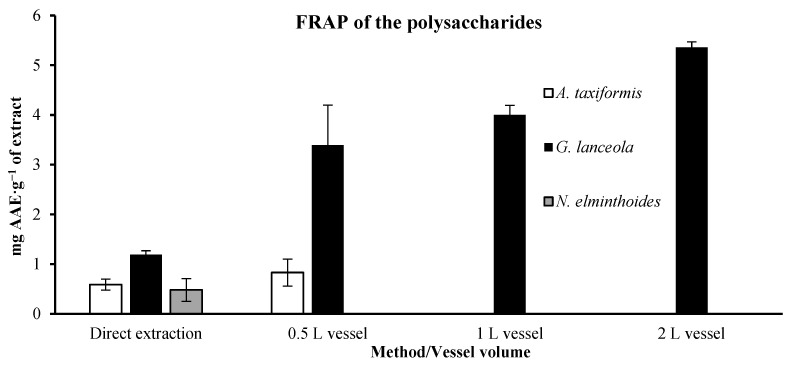
Ferric Reducing Antioxidant Power (FRAP) of polysaccharide extracts obtained through direct extraction and sequential biorefinery processing at different vessel volumes. Results are expressed as mg of ascorbic acid equivalents per gram of extract. (mg AAE·g^−1^ extract). Bars represent mean values ± SD of independent measurements.

The FRAP results for polysaccharide extracts are presented in Figure 10. FRAP values, 0.59 ± 0.11 mg AAE·g^−1^ for *A. taxiformis* were again lower in the direct extract than in the extracts of residual biomass obtained in the 0.5 L vessel, 0.83 ± 0.27 mg AAE·g^−1^ The direct extract of *G. lanceola*, shows a lower value, 1.19 ± 0.08 mg AAE·g^−1^, than the extracts from the residual biomass, which increased proportionally with the volume of the vessel used, varying from 3.39 ± 0.81 (0.5 L vessel) to 5.36 ± 0.11 mg AAE·g^−1^ (2 L vessel). Only the FRAP value of the direct extraction of *N. elminthoides* was determined, as no polysaccharides could be recovered from the residual biomass after the preceding protein and ethanolic extractions. However, the direct extraction presented a very low FRAP value, 0.48 ± 0.23 mg AAE.g^−1^.

#### 2.1.4. Ferrous Ion Chelating (FIC)

**Figure 11:**
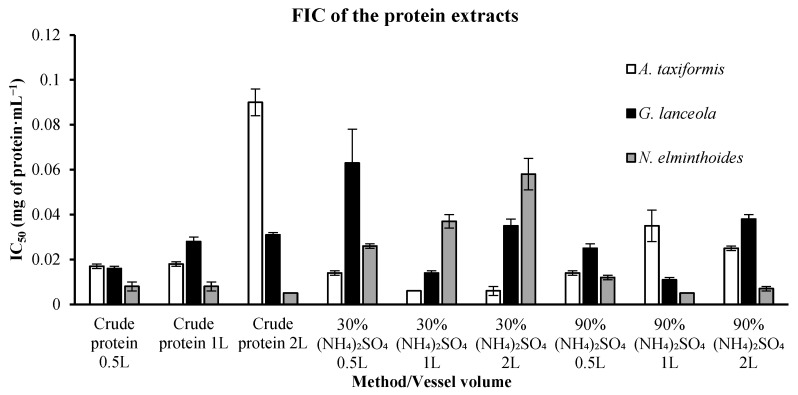
Ferrous ion chelating (FIC) activity of protein-derived extracts obtained through direct extraction and sequential biorefinery processing at different vessel volumes. IC_50_ values (mg·mL^−1^), representing the concentration of soluble protein required to achieve 50% chelation activity, are shown for each extraction condition. Bars represent mean values ± SD of independent measurements. EDTA, used as a positive control, exhibited an IC_50_ value of 0.022738 mg·mL^−1^.

FIC IC_50_ values for protein extracts are presented in Figure 11. It could be observed that lower IC_50_ values for *A. taxiformis* were obtained in the 30% (NH_4_)_2_SO_4_ protein-rich extracts. The FIC values varied between 0.006 ± 0.002 mg of protein·mL^−1^ in the 30% (NH_4_)_2_SO_4_ and 0.090 ± 0.006 mg of protein·mL^−1^ in the crude extracts. This was not the case for *G. lanceola*, for which the lowest value, 0.011 ± 0.001 mg of protein·mL^−1^, was determined with the 90% (NH_4_)_2_SO_4_, and the highest, 0.063 ± 0.015 mg of protein·mL^−1^, with the 30% (NH_4_)_2_SO_4_. The lower values of IC_50_ for the *N. elminthoides* were found in two distinct situations, on the crude extract and 90% (NH_4_)_2_SO_4_, both presenting 0.005 ± 0 mg of protein·mL^−1^. The highest FIC IC_50_ value, 0.058 ± 0.007 mg of protein·mL^−1^, was determined in the 30% (NH_4_)_2_SO_4_ precipitation extracts.

**Figure 12:**
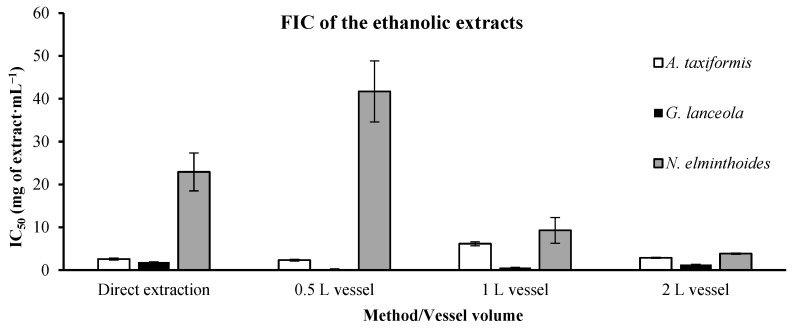
Ferrous ion chelating (FIC) activity of ethanolic extracts obtained through direct extraction and sequential biorefinery processing at different vessel volumes. IC_50_ values (mg extract·mL^−1^), representing the concentration of extract required to achieve 50% chelation activity, are shown for each extraction condition. Bars represent mean values ± SD of independent measurements. EDTA, used as a positive control, exhibited an IC_50_ value of 0.022738 mg·mL^−1^.

Considering the ethanolic extracts (Figure 12), *G. lanceola* shows the lowest extract concentration, 0.258 ± 0.069 mg of extract·mL^−1^, needed to achieve 50% inhibition of ferrous radicals with the biorefinery-derived extract performed with the 0.5 L vessel. *N. elminthoides* extracts demonstrated better IC_50_ values with the integrated fractionation strategy scale-up, which came from 41.737 ± 7.130 (0.5 L vessel) to just 3.853 ± 0.125 mg of extract·mL^−1^ (2 L vessel).

**Figure 13:**
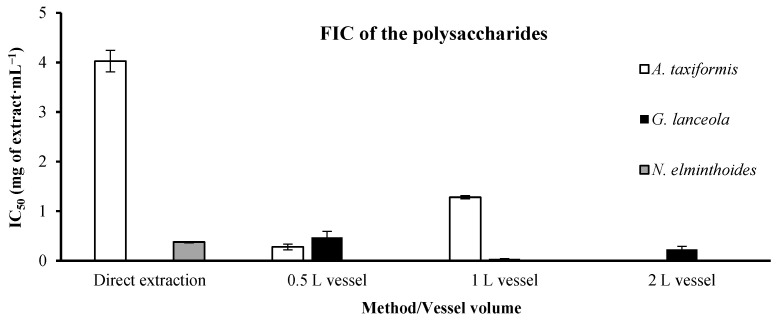
Ferrous ion chelating (FIC) activity of polysaccharide extracts obtained through direct extraction and sequential biorefinery processing at different vessel volumes. IC_50_ values (mg extract·mL^−1^), representing the concentration of extract required to achieve 50% chelation activity, are shown for each extraction condition. Bars represent mean values ± SD of independent measurements. EDTA, used as a positive control, exhibited an IC_50_ value of 0.022738 mg·mL^−1^.

The FIC results of polysaccharide extracts are shown in Figure 13. The *A. taxiformis* extracts have a lower IC_50_ in the biorefinery extracts, 0.27 ± 0.056 mg of extract·mL^−1^, than the direct extract, showing 4.026 ± 0.217 mg of extract·mL^−1^. The *G. lanceola* extracts did not show a linear tendency when considering the biorefinery strategy but presented a very low IC_50_ within the 1 L vessel, 0.035 ± 0.007 mg of extract·mL^−1^. For *N. elminthoides,* only the IC_50_ for the direct extraction was assessed, 0.376 ± 0.012 mg of extract·mL^−1^, due to the impossibility of obtaining polysaccharide extract from the biorefinery residual biomass.

### 2.2. Bioactivity Assays

#### 2.2.1. Anti-Cholinesterase Activity

**Figure 14:**
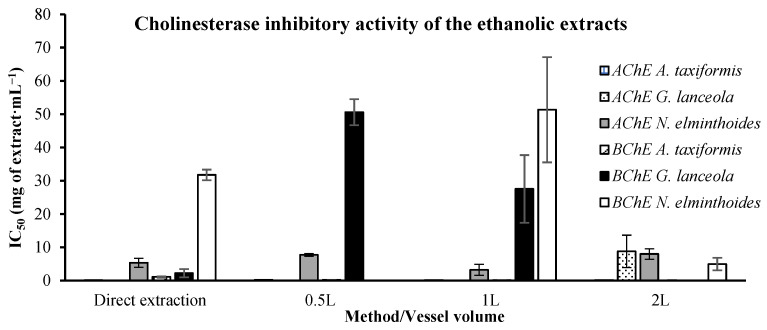
In vitro cholinesterase inhibitory activity of ethanolic extracts obtained from red macroalgae through direct extraction and sequential biorefinery processing at different vessel volumes. IC_50_ values (mg·mL^−1^), representing the concentration of extract required to inhibit 50% of enzyme activity, are shown for acetylcholinesterase (AChE) and butyrylcholinesterase (BChE). Bars represent mean values ± SD of independent measurements. Donepezil standard presented an IC_50_ of 0.06753 × 10^−3^ mg·mL^−1^ for AChE and 3.123 × 10^−3^ mg·mL^−1^ for BChE.

Acetylcholinesterase and butyrylcholinesterase activities were assessed in the ethanolic extracts, comparing direct and biorefinery extracts (Figure 14). The acetylcholinesterase activity demonstrated a lower IC_50_ value (0.137 ± 0.018 mg·mL^−1^) in the direct extract of *A. taxiformis* than the extracts derived from the same macroalgae applying the integrated fractionation strategy. This behavior was also observed for *G. lanceola*, for which the direct extract rendered an IC_50_ value as low as 0.077 ± 0.024 mg·mL^−1^. Donepezil, a standard that was used to compare to our extracts’ activity, a clinically approved cholinesterase inhibitor, presented an IC_50_ of 0.06753 ×10^−3^ mg·mL^−1^.

The butyrylcholinesterase activity (Figure 14) still shows the same tendency with the *G. lanceola* extracts, with the direct extract presenting a lower value (2.256 ± 1.219 mg·mL^−1^) than the biorefinery-derived extracts (27.536 ± 10.172 mg·mL^−1^) but not for *A. taxiformis*. Its extracts showed the opposite trend in the butyrylcholinesterase assay, as the direct extract required a higher concentration (1.109 ± 0.235 mg·mL^−1^) to achieve 50% inhibition, whereas the biorefinery-derived extracts required lower concentrations (as low as 0.112 ± 0.036 mg·mL^−1^). Donepezil presented a much lower IC_50_ value, 3.123 ×10^−3^ mg·mL^−1^.

The relatively high standard deviations observed in some IC_50_ values derived from acetylcholinesterase and butyrylcholinesterase activities, particularly for biorefinery-derived extracts at higher processing volumes, may reflect variability inherent to dose–response curve fitting when inhibitory activity approaches the upper concentration range tested. In these cases, partial inhibition within the tested concentration interval can increase uncertainty in nonlinear regression-derived IC_50_ values.

#### 2.2.2. Anti-α-Glucosidase

**Figure 15:**
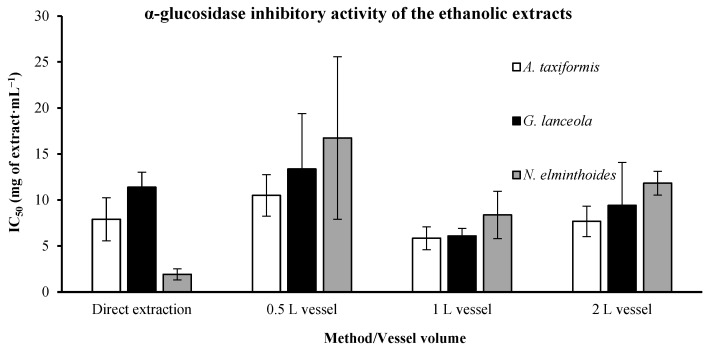
In vitro α-glucosidase inhibitory activity of ethanolic extracts obtained from red macroalgae through direct extraction and sequential biorefinery processing at different vessel volumes. IC_50_ values (mg·mL^−1^), representing the concentration of extract required to inhibit 50% of enzymatic activity, are shown for each extraction condition. Bars represent mean values ± SD of independent measurements.

The IC_50_ determined for anti-α-glucosidase (Figure 15) varied from 1.914 ± 0.597 mg·mL^−1^ observed in the direct extraction and 16.736 ± 8.827 mg·mL^−1^, derived from the 0.5 L vessel, both from the *N. elminthoides*. The direct extraction does not provide lower IC_50_ values than the scale-up process in *A. taxiformis* and *G. lanceola*. Acarbose was used as a comparison standard, demonstrating a low IC_50_, 0.3127 mg·mL^−1^.

Similarly, greater dispersion observed in a limited α-glucosidase IC_50_ values may be attributed to reduced inhibition potency near the assay detection threshold, where small absorbance variations can significantly influence curve-fitting parameters.

### 2.3. Statistical Analysis

Spearman correlation analyses were conducted to explore the relationships among various antioxidants and bioactive assays performed on protein, ethanol, and polysaccharide extracts. The correlation matrix for the protein extracts (Table A1) revealed a significant relationship between ORAC and FRAP (r = 0.605, *p* ≤ 0.01). Also, significant correlations within the bioactive and polysaccharides extracts were also observed (Table A2). FRAP values of the polysaccharide fractions were negatively correlated with FIC IC_50_ values of ethanolic extracts (r = −0.848, *p* ≤ 0.01). Again, within the polysaccharides, FRAP values were determined to be directly correlated with DPPH values (r = 0.752, *p* ≤ 0.01).

**Figure 16:**
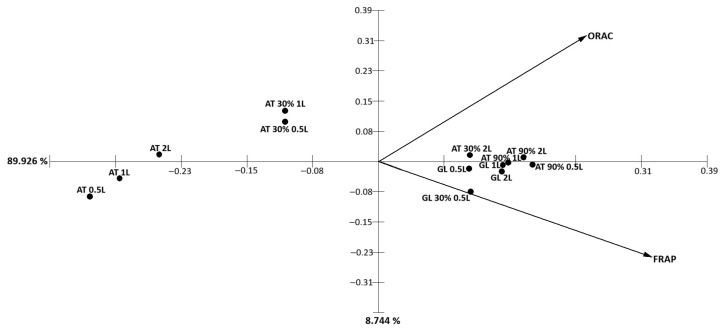
Principal component analysis (PCA) of antioxidant parameters obtained for protein extracts from red macroalgae subjected to direct extraction and sequential ammonium sulfate fractionation. PC1 and PC2 explained 89.9% and 8.7% of the total variance, respectively. The distribution of samples along PC1 was mainly influenced by ORAC and FRAP values, which loaded in opposite directions, highlighting fraction-dependent differences in dominant antioxidant mechanisms.

Principal component analysis (PCA) of the antioxidant parameters obtained for protein extracts revealed that PC1 accounted for 89.9% of the total variance, while PC2 explained 8.7% (Figure 16). The separation of samples along PC1 was mainly driven by ORAC and FRAP values, which loaded in opposite directions. Crude protein extracts clustered on the negative side of PC1, whereas ammonium sulfate-fractionated extracts (30% and 90%) were positioned toward the positive axis. This distribution indicates that protein fractionation markedly influenced the dominant antioxidant mechanism expressed by the extracts.

**Figure 17:**
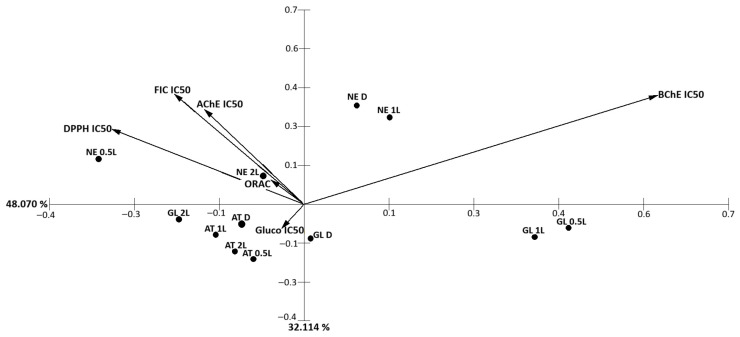
Principal component analysis (PCA) of antioxidants and enzyme inhibitory parameters obtained for ethanolic extracts from red macroalgae subjected to direct extraction and sequential biorefinery processing. PC1 and PC2 explained 48.1% and 32.1% of the total variance, respectively (80.2% cumulative). Separation along PC1 was mainly influenced by BChE IC_50_, while antioxidant-related parameters (DPPH IC_50_, FIC IC_50_, ORAC) and AChE IC_50_ contributed to clustering on the opposite axis. Gluco IC_50_ represents α-glucosidase inhibition.

Principal component analysis (PCA) of ethanolic extracts explained 80.2% of the total variance, with PC1 and PC2 accounting for 48.1% and 32.1%, respectively (Figure 17). PC1 was primarily driven by BChE IC_50_, which showed a strong positive loading, clearly separating *G. lanceola* samples on the positive axis. In contrast, antioxidant-related parameters (DPPH IC_50_, FIC IC_50_, and ORAC) as well as AChE IC_50_ loaded negatively along PC1, contributing to the clustering of *N. elminthoides* and *A. taxiformis* extracts. PC2 was mainly influenced by α-glucosidase IC_50_ and AChE IC_50_, indicating a secondary separation associated with metabolism-related enzyme inhibition.

**Figure 18:**
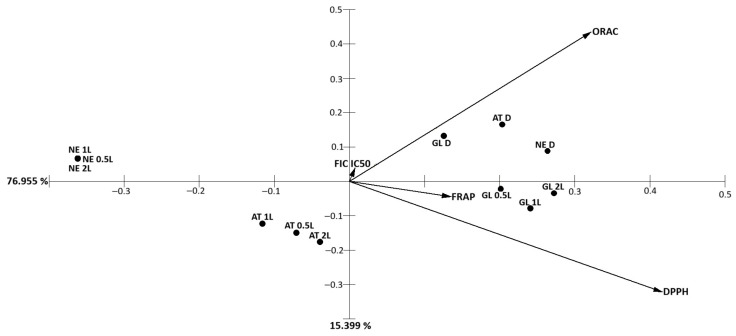
Principal component analysis (PCA) of antioxidant parameters obtained for polysaccharide extracts from red macroalgae subjected to direct extraction and sequential biorefinery processing. PC1 and PC2 explained 77.0% and 15.4% of the total variance, respectively (92.4% cumulative). ORAC and DPPH were the main contributors to sample separation along PC1, highlighting species-dependent differences in antioxidant performance.

Principal component analysis (PCA) of antioxidant parameters obtained for polysaccharide extracts explained 92.4% of the total variance, with PC1 and PC2 accounting for 77.0% and 15.4%, respectively (Figure 18). PC1 was predominantly driven by ORAC and DPPH values, which loaded in opposite directions, while FRAP showed a moderate contribution along the positive axis. *G. lanceola* extracts clustered on the positive side of PC1, associated with higher ORAC and FRAP responses, whereas *N. elminthoides* samples grouped on the negative axis, reflecting lower antioxidant activity. *A. taxiformis* extracts occupied an intermediate position, indicating a distinct but moderate antioxidant profile.

## 3. Discussion

Although the sampled biomass may not capture the full intra-specific physiological variability, it is considered representative of each species within the defined spatial and temporal context of collection. Therefore, the comparisons presented herein refer to biomass-level responses under specific environmental conditions rather than generalized species-wide biochemical characteristics. Among the studied macroalgae, *N. elminthoides* consistently exhibited higher ORAC values compared to *A. taxiformis* and *G. lanceola*, indicating a stronger capacity to scavenge peroxyl radicals through hydrogen atom transfer (HAT) mechanisms. Similar antioxidant behavior has been reported for protein extracts from *Ulva* sp. and *Gracilaria* sp., where antioxidant activity was associated with the presence of phenolic residues bound to proteins and with specific amino acids, such as tryptophan, tyrosine, and methionine, which may become more bioaccessible during gastrointestinal digestion [25]. These features are particularly relevant in a functional food context, as they suggest that macroalgal proteins may contribute to antioxidant protection following dietary intake. For *A. taxiformis* and *N. elminthoides*, ORAC values increased from direct extraction to biorefinery-derived residue extracts obtained at smaller processing volumes (0.5 L), indicating that sequential processing can preserve or enhance antioxidant functionality. However, a consistent decrease in ORAC values was observed as vessel volume increased to 2 L, assessing *A. taxiformis* and *N. elminthoides*, highlighting the sensitivity of HAT-based antioxidant capacity to scale-up conditions. When comparing extract types, ethanolic extracts generally showed higher ORAC values than polysaccharide fractions, likely reflecting their higher phenolic content. The higher antioxidant capacity observed in ethanolic extracts is likely associated with the presence of low- to medium-molecular-weight phenolic compounds typical of Rhodophyta, including bromophenols and phenolic acids [26,27]. These compounds contain hydroxyl groups capable of donating hydrogen atoms (HAT mechanism) and electrons (SET mechanism), thereby explaining the parallel trends observed in ORAC and FRAP assays [28]. In addition, conjugated aromatic structures may stabilize radical intermediates, enhancing radical scavenging efficiency [29]. This distinction underscores the relevance of phenolic-rich fractions as functional food ingredients with enhanced radical scavenging capacity.

The DPPH scavenging activity observed in the present study is comparable to that reported for red macroalgae collected from the north and east coasts of Gran Canaria, where ethanolic extracts exhibited IC_50_ values within a similar range [30]. Such consistency across geographic regions supports the robustness of red macroalgae as dietary sources of radical scavenging compounds. The evaluation of DPPH inhibition at a standardized concentration (150 µg·mL^−1^) allowed a reliable comparison among extracts, as this concentration lies within the linear response range of the assay. The higher inhibition percentages observed in certain direct extracts highlight the contribution of readily extractable antioxidants, which may be particularly relevant for minimally processed functional food formulations.

FRAP values obtained in this study are consistent with previous reports for red macroalgae from the Madeira Archipelago and other Atlantic regions [30,31]. Differences among species and extraction methods likely reflect variations in phenolic composition and solvent polarity. While ethanolic and polysaccharide extracts displayed comparable single electron transfer (SET) capacity, polysaccharide fractions generally exhibited lower FRAP values, suggesting a more limited contribution to reducing power but not excluding their relevance as functional food components. The distinctive environmental conditions of the Madeira Archipelago, characterized by high solar irradiance, oligotrophic waters, and strong hydrodynamic exposure, may promote the biosynthesis of protective secondary metabolites. Such stress-adaptive responses have been associated with increased phenolic and antioxidant production in marine macroalgae from high-irradiation Atlantic regions [7].

Within the FIC evaluation, biorefinery-derived extracts generally showed lower IC_50_ values than direct extracts, indicating enhanced metal-chelating capacity, although this effect was negatively influenced by increasing processing scale. In contrast, *N. elminthoides* extracts exhibited improved chelation efficiency with scale-up, suggesting species-specific responses to processing conditions. Notably, polysaccharide fractions from *A. taxiformis* and *G. lanceola* demonstrated stronger iron-chelating activity than ethanolic extracts, supporting their potential role as dietary antioxidants that limit metal-catalyzed oxidative reactions in food systems and the gastrointestinal tract. This higher iron-chelating capacity may be related to the presence of sulfated galactans and negatively charged functional groups (e.g., sulfate and carboxyl groups), which can coordinate Fe^2+^ ions and limit metal-catalyzed oxidative reactions [32]. The degree of sulfation and molecular weight distribution are known to influence chelation efficiency, suggesting that structural features of these red macroalgal polysaccharides contribute to their functional properties [15]. The decrease in antioxidant activity observed at larger processing volumes (2 L) may reflect partial degradation or oxidation of sensitive bioactive compounds during extended extraction cycles and increased exposure to oxygen [2]. Although temperature and pressure were controlled, scale-up may alter mass transfer dynamics and increase residence time, potentially promoting structural modification of phenolic compounds or partial denaturation of bioactive proteins [33,34]. Such effects have been reported in scaled extraction systems and highlight the importance of optimizing process parameters to preserve functional integrity.

Ethanolic extracts displayed a higher inhibitory capacity toward acetylcholinesterase (AChE) than butyrylcholinesterase (BuChE), suggesting selectivity toward mechanisms associated with cholinergic function. The reduced activity observed in some biorefinery-derived extracts indicates that certain bioactive components may be partially removed during prior extraction steps. Although the inhibitory potency was markedly lower than that of donepezil, a pharmaceutical standard, the observed activities remain relevant from a functional food perspective, where mild and sustained modulation of enzyme activity through diet is desirable rather than acute pharmacological inhibition.

α-Glucosidase inhibition varied according to species and extraction scale, with lower IC_50_ values frequently observed in extracts derived from intermediate processing volumes (1 L). While acarbose exhibited significantly stronger inhibition, the activities detected in macroalgal extracts suggest a potential role in supporting postprandial glycemic control when incorporated into functional food matrices.

The convergence of antioxidant, cholinesterase inhibitory, and α-glucosidase inhibitory activities observed in selected extracts supports a mechanistically integrated model, where modulation of oxidative stress, cholinergic signaling, and postprandial glycemic response may collectively contribute to neuroprotective and metabolic health support. Rather than representing isolated bioactivities, these functions reflect interconnected biochemical pathways relevant to diet-based strategies targeting cognitive decline. The coexistence of antioxidant and cholinesterase inhibitory activities observed in certain extracts is particularly relevant in the context of Alzheimer’s disease, where oxidative imbalance and cholinergic dysfunction are mechanistically interconnected. Antioxidant compounds may mitigate ROS-mediated neuronal damage, while mild cholinesterase inhibition may support synaptic acetylcholine availability [35,36]. Collectively, these findings align with the emerging concept that dietary bioactives capable of simultaneously modulating redox balance, enzymatic glycemic control, and cholinergic function may offer complementary benefits in mitigating interconnected metabolic and neurodegenerative processes.

Principal component analysis (PCA) revealed clear fraction-dependent bioactivity patterns among protein, ethanolic, and polysaccharide extracts. In protein fractions, antioxidant performance was mainly structured by contrasting ORAC and FRAP contributions, indicating differential expression of hydrogen atom transfer and single electron transfer mechanisms following ammonium sulfate fractionation. Ethanolic extracts displayed a more complex distribution, where antioxidant parameters and enzyme inhibitory activities jointly contributed to sample separation, particularly through BChE IC_50_ loadings, highlighting their multifunctional profile. In contrast, polysaccharide extracts were primarily driven by radical scavenging parameters (ORAC and DPPH), suggesting a more consistent antioxidant-centered behavior. Collectively, these multivariate patterns demonstrate that sequential biorefinery processing differentially modulates the dominant mechanistic pathways of each fraction, supporting a modular and complementary contribution of red macroalgal extracts within the proposed neuro–metabolic framework.

Spearman correlation analyses revealed a significant positive association between ORAC and FRAP values in protein extracts, indicating that redox-active compounds contribute simultaneously to hydrogen atom transfer (HAT) and single electron transfer (SET) antioxidant mechanisms. This relationship highlights the multifactorial nature of antioxidant functionality in protein-rich macroalgal extracts, where specific constituents such as phycobiliproteins and associated phenolic residues may exert complementary antioxidant effects relevant to dietary antioxidant protection. In polysaccharide-rich fractions, FRAP values were strongly and inversely correlated with the IC_50_ values obtained for ferrous ion chelating (FIC) activity in ethanol extracts, indicating that higher reducing power is associated with greater metal-chelating efficiency. Interestingly, species exhibiting higher reducing power in polysaccharide fractions tended to display stronger iron-chelating capacity in phenolic-rich extracts. This relationship may reflect an integrated biochemical strategy in which both redox-active compounds and metal-binding constituents contribute to oxidative stress mitigation. Such complementary behavior supports the hypothesis that antioxidant functionality in red macroalgae is not confined to a single molecular class but rather distributed across structurally distinct fractions [1]. A similar relationship between reducing power and chelation capacity has been previously reported for macroalgal extracts obtained with 50% methanol [31], reinforcing the consistency of this antioxidant pattern across extraction strategies. Additionally, a strong positive correlation was observed between FRAP values and DPPH radical scavenging activity within polysaccharide extracts, consistent with the presence of hydrophilic antioxidants capable of both electron donation and hydrogen atom transfer. These properties support the potential role of macroalgal polysaccharides as functional ingredients contributing to oxidative balance in food systems and during digestion. Collectively, these correlations emphasize the biochemical complexity and extract-specific bioactivity of macroalgal fractions. Ethanol extracts appear to concentrate multifunctional compounds with broad antioxidant potential, while polysaccharide fractions may harbor distinct antioxidant agents with relevance for functional food applications.

Although the present study provides a comprehensive in vitro assessment of anti-oxidant and enzyme inhibitory activities, further research is necessary to validate the physiological relevance of these findings. Future studies should investigate bioaccessibility and bioavailability using simulated gastrointestinal digestion models, followed by cellular assays to evaluate intracellular antioxidant effects and modulation of cholinergic and metabolic pathways. In vivo studies, particularly in models of metabolic dysregulation or neurodegeneration, would be essential to confirm the functional implications suggested by the current data. Additionally, structural characterization of the bioactive fractions would further clarify structure–function relationships and support targeted biorefinery optimization.

## 4. Materials and Methods

### 4.1. Macroalgae from Madeira Archipelago

Specimens of *Asparagopsis taxiformis* (Delile) Trevisan (32.647155–16.824185), *Grateloupia lanceola* (J. Agardh) J. Agardh (32.659932–17.053886), and *Nemalion elminthoides* (Velley) Batters (32.734273–16.745741) were collected at the end of spring (May 2022) in the Madeira Archipelago (Portugal, NE Atlantic Ocean). Approximately 5 kg (fresh weight) of each species were harvested at a maximum depth of 10 m. *A. taxiformis* and *N. elminthoides* were collected from subtidal rocky shores, whereas *G. lanceola* was collected from offshore *Sparus aurata* aquaculture cages located more than 600 m from the coastline. Immediately after collection, samples were transported to the laboratory in seawater under refrigerated conditions (4 °C) and gently rinsed with filtered distilled water to remove salts, sand, and epiphytes. The biomass was vacuum-packed and stored at −35 °C until further processing. Before extraction procedures, frozen samples were thawed at 4 °C. For biorefinery experiments involving dried biomass, solid residues were oven-dried at 40 °C until constant weight and subsequently milled to obtain a homogeneous powder (particle size < 1 mm). Dried samples were stored in airtight containers protected from light and humidity at room temperature until analysis. Three independent biological replicates were considered for each species, consisting of biomass collected from spatially separated individuals within each sampling site.

### 4.2. Biorefinery

All biorefinery extractions were performed under controlled and reproducible operational parameters. The direct extraction (DE) procedure was conducted exclusively at 0.5 L, serving as a reference condition. In contrast, the sequential biorefinery process was evaluated at three operational volumes (0.5, 1, and 2 L) to assess scale-up effects while maintaining constant extraction parameters. The initial protein extraction was performed at 4 °C using a biomass-to-solvent ratio of 110 g fresh weight per L of phosphate buffer (0.1 M, pH 6.8), with 18 extraction cycles in a Timatic Micro-DP-LT.0.5–1–2 system (Technolab, Meda, Italy), employing a compression time of 3 min and decompression time of 6 min (total extraction time: 162 min). Ethanol extraction (96%) was subsequently conducted at 22 °C for 18 cycles under identical pressure conditions (8.5 bar). Polysaccharides were extracted from the residual biomass using distilled water at 85 °C for 4 h under continuous stirring. Centrifugation steps were performed at 15,000× *g* for 15 min at 4 °C for protein fractions and at 7200× *g* for 10 min at 21 °C for polysaccharide precipitation. For the biorefinery approach, the solid–liquid ratio and extraction time were maintained constant across scale-up volumes (0.5, 1, and 2 L), ensuring process comparability while isolating the effect of operational scale.

Further information on optimization of protein extraction, as well as the determination of protein and phycoerythrin content, total phenolic content (TPC), and the production of ethanolic and polysaccharide extracts, was previously described in detail by Nunes et al. (2026) [37].

### 4.3. Antioxidant Activities

#### 4.3.1. Oxygen Radical Absorbance Capacity (ORAC)

ORAC assay was performed [38] adding phosphate-buffered solution (0.01 M pH 7.4) to a black microplate (Thermo Scientific™ Nunc™ F96 MicroWell™ Black Polystyrene Plate; Thermo Fisher Scientific, Roskilde, Denmark), a fluorescein solution (1µM) and 2,2′-azobis(2-methylpropionamidine) dihydrochloride (AAPH) (Sigma-Aldrich, St. Louis, MO, USA). A positive control was used by integrating Trolox (Sigma-Aldrich, St. Louis, MO, USA) as an antioxidant standard. A negative control is implemented by not adding the sample or Trolox to the mixture of fluorescein and AAPH buffer. The microplate was incubated at 37 °C for 30 min and the fluorescence was read, exciting at a wavelength of 485 nm and detecting the emission at 518 nm at 30 s intervals for 90 min using a Varioskan LUX microplate reader (Thermo Fisher Scientific, Vantaa, Finland). Data acquisition and analysis were performed using SkanIt Software version 7.1 (Thermo Fisher Scientific, Vantaa, Finland). The extracts were tested at ten concentration points (0.02–20 mg·mL^−1^) to cover the full response range of the scavenging effect. All measurements were performed in triplicate. The results were calculated and expressed as micromoles of Trolox Equivalents per gram of protein in the extract (TE µM·g^−1^ protein), using 10 concentrations (0–212 µM) of Trolox.

#### 4.3.2. Free Radical Scavenging Assay (DPPH)

The DPPH assay uses a stable radical (2,2-diphenyl-1-picrylhydrazyl, Thermo Fisher Scientific, Waltham, MA, USA) to assess the antioxidant capacity of extracts [39,40]. A freshly prepared solution (0.16 mM in methanol) was added to the macroalgae extract in a transparent 96-well microplate. After incubation at room temperature in the dark for 30 min, it was measured at 517 nm using a Varioskan LUX microplate reader (Thermo Fisher Scientific, Vantaa, Finland). Data acquisition and analysis were performed using SkanIt Software version 7.1 (Thermo Fisher Scientific, Vantaa, Finland). The DPPH radical scavenging capacity is calculated from Equation (1), described below. The extracts were tested at ten concentration points (0.04–20 mg·mL^−1^) to cover the full response range to determine the extract concentration that presented 50% of inhibition. All measurements were performed in triplicate. The results were calculated and expressed as % of inhibition and IC_50_. Ascorbic acid (Sigma-Aldrich, St. Louis, MO, USA) was used as a positive standard, with 6 concentration points (0–12.5 µg·mL^−1^), which were assessed.

Equation (1):

(1) $$\%\;inhibition=\left[1-\frac{Asample\;or\;positive\;Std}{Acontrol}\right]\times 100$$

*Asample*—Resulting absorbance of the reaction between the extract or positive standard (ascorbic acid) and DPPH.

*Acontrol*—An equal amount of methanol and DPPH without a sample.

#### 4.3.3. Ferric Reducing Antioxidant Power (FRAP)

FRAP assay [41,42] was performed by adding 625 µL of a phosphate buffer (0.2 M, pH 6.6) to an equal volume of potassium hexacyanoferrate at 1% (*w*/*v*). The solution was blended with 250 µL of extract and incubated in a water bath at 50 °C for 20 min. Subsequently, 625 µL of trichloroacetic acid (TCA) at 10% (*w*/*v*), 625 µL of water and 125 µL of ferric chloride hexahydrate 0.1% (*w*/*v*) were mixed to 625 µL of the reaction mixture. This was incubated in the dark, at room temperature, for 10 min to allow color development. The absorbance was assessed at 700 nm in a PC-2401 spectrophotometer (Shimadzu Corporation, Kyoto, Japan). Values were expressed as mg of ascorbic acid equivalents (AAE) per g of dry weight (dw) sample (mg AAE·g^−1^ sample dw). The calibration equation for ascorbic acid was assessed with 6 concentration points (10–125 µg·mL^−1^) and the equation obtained was Y = 0.0095x − 0.0078 (R^2^ = 0.9964). All reagents were obtained from Sigma-Aldrich (St. Louis, MO, USA).

#### 4.3.4. Ferrous Ion Chelating (FIC)

FIC assay was performed [43,44] using EDTA as a positive control/standard with 6 concentration points (0–40 µg·mL^−1^) covering from 0 to 98% of chelating ability. The assay was executed in a 96-well microplate, adding equal amounts of ferrous sulphate (FeSO_4_.7H_2_O, 0.1 mM) to the sample or standard. After mixing, ferrozine (0.25 mM) was added to the mixture and the microplate was incubated for 10 min. All reagents were obtained from Sigma-Aldrich (St. Louis, MO, USA). Afterwards, the reaction was measured at 562 nm with a Varioskan LUX microplate reader (Thermo Fisher Scientific, Vantaa, Finland). Data acquisition and analysis were performed using SkanIt Software version 7.1 (Thermo Fisher Scientific, Vantaa, Finland). The percentage (%) of chelating ability was calculated from Equation (2), described below. The extracts were tested at six concentration points (0–2 mg·mL^−1^) to cover the full response range and determine the extract concentration that presented 50% of chelating ability. All measurements were performed in triplicate. The results were calculated and expressed as IC_50_.

Equation (2):

(2) $$\%\;chelating\;ability=\left[\frac{Acontrol-Asample}{Acontrol}\right]\times 100$$

*Acontrol*—Mixture of water, ferrous sulphate and ferrozine.

*Asample*—Mixture of sample or standard, ferrous sulphate and ferrozine.

### 4.4. Bioactivity Assays

#### 4.4.1. Anti-Cholinesterase Activity

**Figure A1:**
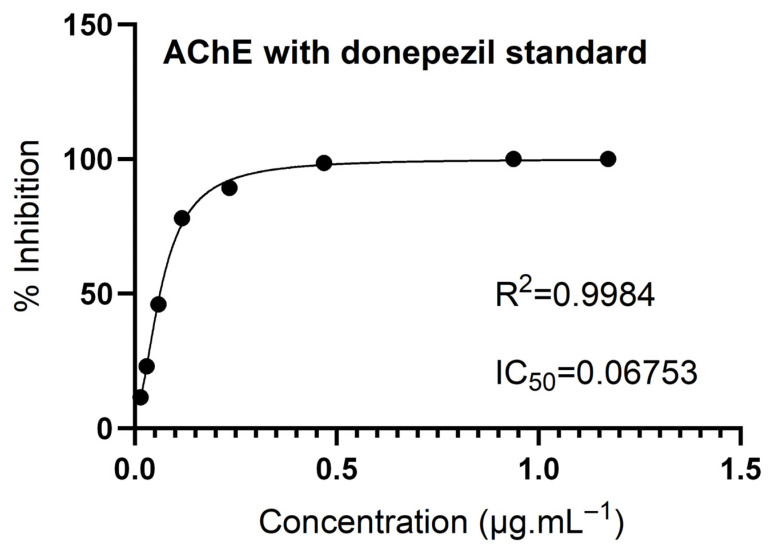
Inhibitory effect of donepezil on acetylcholinesterase (AChE) activity. Percentage of inhibition (%) is plotted as a function of concentration (µg·mL^−1^). Data points represent mean values of independent measurements, and the curve was fitted using nonlinear regression based on a four-parameter logistic (4PL) model. Goodness-of-fit parameters indicated an excellent model fit, with a well-defined IC_50_ value of 0.0675 µg·mL^−1^ (95% CI: 0.0561–0.0780 µg·mL^−1^). Donepezil was tested over a concentration range sufficient to define both upper and lower asymptotes of the dose–response curve.

**Figure A2:**
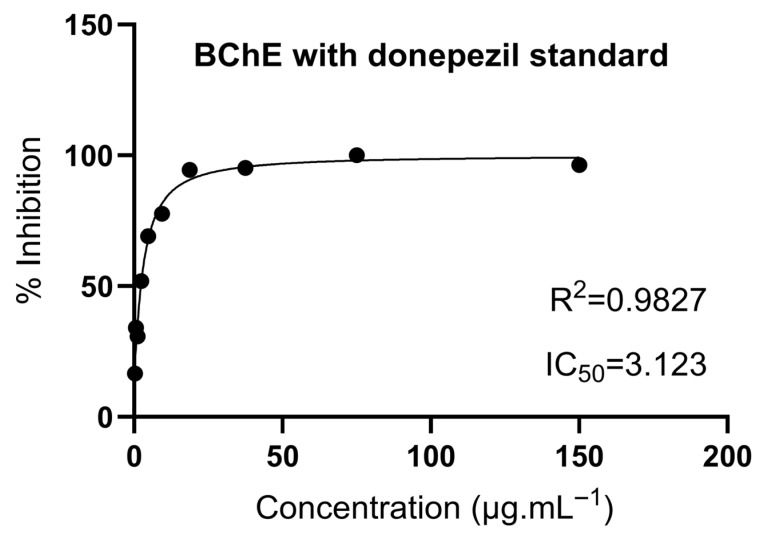
Inhibitory effect of donepezil on butyrylcholinesterase (BChE) activity. Percentage of inhibition (%) is plotted as a function of concentration (µg·mL^−1^). Data points represent mean values of independent measurements, and the curve was fitted using nonlinear regression based on a four-parameter logistic (4PL) model. Goodness-of-fit parameters indicated an acceptable model fit (R^2^ = 0.9827), yielding an IC_50_ value of 3.123 µg·mL^−1^ (95% CI: 0.495–5.281 µg·mL^−1^). Donepezil was tested over a concentration range sufficient to define the dose–response trend.

Anti-acetylcholinesterase (AChE) and anti-butyrylcholinesterase activities (BuChE) were performed on the phenolic (ethanolic) extracts using transparent microplates with serial dilutions or positive standard (Donepezil) in phosphate buffer (100 mM, pH 8) [45,46]. Subsequently, 0.25 U.mL^−1^ of acetylcholinesterase or butyrylcholinesterase was added and left to incubate for 5 min. The reaction started by the addition of a substrate mixture, composed of the same volume of 3 mM 5,5′-dithiobis [2-nitrobenzoic acid] (DTNB) and 75 mM acetylthiocholine iodide (ATChI) for AChE or butyrylthiocholine iodide (BuTChI) for BChE. All reagents were obtained from Sigma-Aldrich (St. Louis, MO, USA). The absorbance was read at 415 nm in a microplate reader at different times, 0, 150, 300, and 450 s Varioskan LUX microplate reader (Thermo Fisher Scientific, Vantaa, Finland). Data acquisition and analysis were performed using SkanIt Software version 7.1 (Thermo Fisher Scientific, Vantaa, Finland). Enzyme inhibition was determined as the percentage activity of reaction media containing samples and the activity of the control without inhibitor. Donepezil (0–150 µg·mL^−1^) and the samples (0–4 mg·mL^−1^) were evaluated at ten or more concentration points to ensure adequate coverage. IC_50_ values were subsequently determined from the dose–response curves, represented as Figure A1 for AChE and Figure A2 for BuChE. All measurements were performed in triplicate. The results were calculated and expressed as IC_50_.

#### 4.4.2. Anti-α-Glucosidase

**Figure A3:**
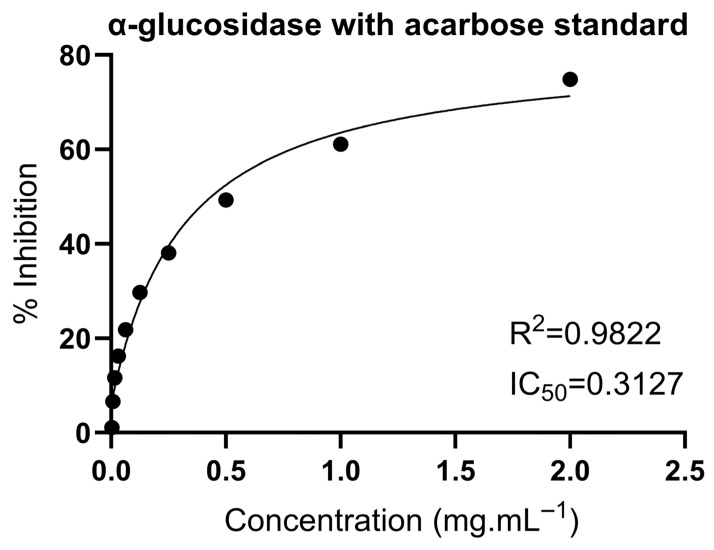
Inhibitory effect of acarbose on α-glucosidase activity. Percentage of inhibition (%) is presented as a function of concentration (mg·mL^−1^). Data points represent mean values of independent measurements, and the curve was fitted using nonlinear regression based on a three-parameter logistic (3PL) model. Goodness-of-fit parameters indicated a satisfactory model fit (R^2^ = 0.9822), yielding an IC_50_ value of 0.3127 mg·mL^−1^ (95% CI: 0.1622–0.6224 mg·mL^−1^). Acarbose was tested over a concentration range sufficient to define the dose–response relationship.

The α-glucosidase inhibition test was performed on the phenolic (ethanolic) extracts and the sample or acarbose standard (positive control) was mixed with phosphate buffer (100 mM pH 6.9) in a transparent microplate for serial dilution. Then, 1 U·mL^−1^ of α-glucosidase solution was added and the microplate was incubated at 25 °C for 10 min [45,47,48,49]. A solution of 5 mM of p-nitrophenyl-α-D-glucopyranoside (PNPG) was added to all the wells and the absorbance was read at 405 nm at different times, 0, 150, 300 and 450 s Varioskan LUX microplate reader (Thermo Fisher Scientific, Vantaa, Finland). Data acquisition and analysis were performed using SkanIt Software version 7.1 (Thermo Fisher Scientific, Vantaa, Finland). Enzyme inhibition was determined as the % activity of the reaction media containing the sample and the activity of the negative control without the sample, with calculations made as presented in Equation (3). Acarbose (0–2 mg·mL^−1^) was evaluated at ten concentration points and the samples (0–2 mg·mL^−1^) were evaluated at six concentration points to ensure adequate coverage. All reagents were obtained from Sigma-Aldrich (St. Louis, MO, USA). IC_50_ values were subsequently determined from the dose–response curves, represented as Figure A3. All measurements were performed in triplicate. The results were calculated and expressed as IC_50_.

Equation (3):

(3) $$\%\alpha -Glucosidase\;inhibition=\frac{∆A\;Negative\;control-∆A\;Sample}{∆A\;Negative\;control}\times 100$$

*Negative control*—Phosphate buffer + 1 U·mL^−1^ α-glucosidase + PNPG.

*Sample*—Sample solution + Phosphate buffer + 1 U·mL^−1^ α-glucosidase + PNPG.

### 4.5. Statistics

Data are expressed as the mean of three independent replicates ± standard deviation. Statistical analyses were performed using GraphPad Prism version 10.5.0 (GraphPad Software, San Diego, CA, USA), IBM SPSS Statistics version 30.0 (IBM, Armonk, NY, USA), and MVSP version 3.12 (Kovach Computing Services, Anglesey, Wales, UK). Assumptions of normality and homogeneity of variances were evaluated using the Shapiro–Wilk and Levene tests, respectively. As the data did not meet these assumptions, even after square root and logarithmic transformations, non-parametric statistical tests were applied. Differences among groups were assessed using the Kruskal–Wallis test. Correlations between antioxidant parameters were evaluated using Spearman’s rank correlation coefficient. Statistical significance was considered at *p* < 0.01.

## 5. Conclusions

The sequential biorefinery approach applied to *A. taxiformis*, *G. lanceola*, and *N. elminthoides* enabled the recovery and characterization of multiple fractions with relevant functional properties for food and nutrition applications. Protein-rich extracts, particularly from *N. elminthoides*, exhibited strong hydrogen atom transfer-based antioxidant capacity, highlighting their potential as functional dietary components. Ethanolic (phenolic-rich) extracts demonstrated broader multifunctional activity, combining radical scavenging, metal chelation, and enzyme inhibition associated with cognitive health support and postprandial glycemic regulation. Polysaccharide fractions, although generally displaying lower overall activity, contributed notably to iron-chelating and reducing capacities, supporting their role as functional ingredients in food formulations. Direct extracts and biorefinery-derived fractions showed distinct functional profiles, with enzyme inhibition assays indicating greater selectivity toward acetylcholinesterase than butyrylcholinesterase, and α-glucosidase inhibition varying according to species and extraction scale. The biorefinery strategy proved sensitive to scale, as smaller processing volumes (0.5 L) generally preserved higher antioxidant functionality, emphasizing the need for careful optimization during scale-up to maintain health-promoting properties. Multivariate analysis suggests that sequential biorefinery processing may influence the dominant bioactivity profiles of protein, ethanolic, and polysaccharide fractions. These patterns indicate potential complementary and mechanism-specific contribution of red macroalgal extracts within the proposed neuro–metabolic modulation framework. However, these associations are based on in vitro assays and correlation analyses and therefore should be interpreted as indicative rather than causative relationships. Optimized biorefinery strategies may benefit from prioritizing controlled residence time, moderate processing volumes, and sequential extraction schemes that preserve phenolic and protein integrity. Future optimization may focus on refining extraction time and oxygen exposure control to further enhance bioactive retention during scale-up. Overall, this study positions Madeira red macroalgae as sustainable sources of multifunctional ingredients capable of modulating interconnected oxidative, cholinergic, and metabolic pathways, while underscoring the importance of optimized biorefinery strategies to preserve mechanistically relevant bioactivity during scale-up.

## Data Availability

The data that support the findings of this study are available from the corresponding author upon reasonable request.

## Abbreviations

The following abbreviations are used in this manuscript:

AAE	Ascorbic Acid Equivalents
AChE	Acetylcholinesterase
AD	Alzheimer disease
AGE	Advanced glycation end-product
ATChI	Acetylthiocholine iodide
BuChE	Butyrylcholinesterase
BuTChI	Butyrylthiocholine iodide
CC BY	Creative Commons Attribution
DPPH	2,2-Diphenyl-1-picrylhydrazyl
DTNB	5,5′-Dithiobis(2-nitrobenzoic acid)
dw	Dry weight
EDTA	Ethylenediaminetetraacetic acid
EtOH	Ethanol
FIC	Ferrous Ion Chelating
FRAP	Ferric Reducing Antioxidant Power
IC_50_	Inhibitory Concentration 50%
nd	Not determined
(NH_4_)_2_SO_4_	Ammonium sulfate
ORAC	Oxygen Radical Absorbance Capacity
PCA	Principal component analysis
PNPG	p-Nitrophenyl-α-D-glucopyranoside
SD	Standard Deviation
SPSS	Statistical Package for the Social Sciences
TCA	Trichloroacetic Acid
Trolox eq.	Trolox equivalents
U	Enzyme unit

## Appendix B

**Table A1:** Spearman correlations between all the parameters assessed for the protein extracts. The statistical analysis was carried out using SPSS 30.0 and all significant results (*p* ≤ 0.01) are marked (*).

	FIC IC_50_ (mg Protein·mL^−1^)	FRAP (mg AAE·g^−1^ Protein)	DPPH IC_50_ (mg Protein·mL^−1^)	ORAC Trolox eq (µM·g^−1^ Protein)
FIC IC_50_ (mg protein·mL^−1^)	1	0.335	0.337	−0.064
FRAP (mg AAE·g^−1^ protein)	0.335	1	0.370	0.605 *
DPPH IC_50_ (mg protein·mL^−1^)	0.337	0.370	1	−0.002
ORAC Trolox eq (µM·g^−1^ protein)	−0.064	0.605 *	−0.002	1

**Table A2:** Spearman correlations between all the parameters assessed for the ethanol and polysaccharides extracts. The statistical analysis was carried out using SPSS 30.0 and all significant results (*p* ≤ 0.01) are marked (*).

	FIC EtOH IC_50_ (mg·mL^−1^)	FIC Poly IC_50_ (mg·mL^−1^)	FRAP EtOH (mg AAE·g^−1^)	FRAP Poly (mg AAE·g^−1^)	DPPH EtOH IC_50_ (mg·mL^−1^)	DPPH Poly (% Capture 150 µg·mL^−1^)	ORAC EtOH (Trolox eq. µM·g^−1^)	ORAC Poly (Trolox eq. µM·g^−1^)	AChE IC_50_ (mg·mL^−1^)	BuChE IC_50_ (mg·mL^−1^)	Gluco IC_50_ (mg·mL^−1^)
FIC EtOH IC_50_ (mg·mL^−1^)	1	0.211	−0.133	−0.848 *	0.62	−0.613	0.168	−0.347	0.538	−0.109	−0.13
FIC Poly IC_50_ (mg·mL^−1^)	0.211	1	−0.469	−0.299	0.578	−0.135	−0.38	−0.135	0.458	−0.61	−0.007
FRAP EtOH (mg AAE·g^−1^)	−0.133	−0.469	1	0.276	−0.523	0.307	0.259	0.178	−0.231	0.082	−0.481
FRAP Poly (mg AAE·g^−1^)	−0.848 *	−0.299	0.276	1	−0.512	0.752 *	0.145	0.608	−0.334	0.076	0.029
DPPH EtOH IC_50_ (mg·mL^−1^)	0.62	0.578	−0.523	−0.512	1	−0.504	−0.116	0.019	0.588	−0.109	0.037
DPPH Poly (% capture 150 µg·mL^−1^)	−0.613	−0.135	0.307	0.752 *	−0.504	1	−0.049	0.571	−0.31	0.046	−0.459
ORAC EtOH (Trolox eq. µM·g^−1^)	0.168	−0.38	0.259	0.145	−0.116	−0.049	1	0.284	0.112	−0.168	0.305
ORAC Poly (Trolox eq. µM·g^−1^)	−0.347	−0.135	0.178	0.608	0.019	0.571	0.284	1	−0.272	0.2	−0.215
AChE IC_50_ (mg·mL^−1^)	0.538	0.458	−0.231	−0.334	0.588	−0.31	0.112	−0.272	1	−0.322	0.2
BuChE IC_50_ (mg·mL^−1^)	−0.109	−0.61	0.082	0.076	−0.109	0.046	−0.168	0.2	−0.322	1	−0.126
Gluco IC_50_ (mg·mL^−1^)	−0.13	−0.007	−0.481	0.029	0.037	−0.459	0.305	−0.215	0.2	−0.126	1
